# Rapid Screening and Quantitative Determination of Active Components in Qing-Hua-Yu-Re-Formula Using UHPLC-Q-TOF/MS and HPLC-UV

**DOI:** 10.1155/2018/8535127

**Published:** 2018-03-11

**Authors:** Xin Shao, Jie Zhao, Xu Wang, Yi Tao

**Affiliations:** ^1^The First Clinical Medical College, Nanjing University of Chinese Medicine, Nanjing 210023, China; ^2^Department of Endocrinology, Nanjing Hospital of Traditional Chinese Medicine, Nanjing 210001, China; ^3^Pharmaceutical Animal Experimental Center, China Pharmaceutical University, Nanjing 210009, China; ^4^School of Pharmacy, Nanjing University of Chinese Medicine, Nanjing 210023, China

## Abstract

Qing-Hua-Yu-Re-Formula (QHYRF), a new herbal preparation, has been extensively used for treating diabetic cardiomyopathy. However, the chemical constituents of QHYRF remain uninvestigated. In the present study, rapid ultrahigh-performance liquid chromatography coupled with quadrupole-time-of-flight mass spectrometry (UHPLC-Q-TOF/MS) was used to qualitatively analyze the components of QHYRF. Qualitative detection was performed on a Kromasil C_18_ column through the gradient elution mode, using acetonitrile-water containing 0.1% formic acid. Twenty-seven compounds were identified or tentatively characterized, including 12 phenolic acids, nine monoterpene glycosides, two flavonoids, three iridoids, and one unknown compound. Among these, six compounds were confirmed by comparing with standards. A high-performance liquid chromatography (HPLC) method was developed to simultaneously determine the following six active components in QHYRF: danshensu, paeoniflorin, acteoside, lithospermic acid, salvianolic acid B, and salvianolic acid C. These HPLC chromatograms were monitored at 254, 280, and 320 nm. The method was well validated with respect to specificity, linearity, limit of detection, limit of quantification, precision, stability, and recovery. The HPLC-UV method was successfully applied to 10 batches of QHYRF.

## 1. Introduction

An increasing number of people have suffered from diabetes in recent years, and cardiovascular complications secondary to diabetes have become the main cause of death in diabetic patients. The incidence of cardiovascular disease in patients with diabetes is 2-3 times higher than that of nondiabetic patients [1]. Diabetic cardiomyopathy (DCM), a specific cardiomyopathy and one of the major cardiac complications in diabetic patients, was found in diabetic patients without significant coronary artery atherosclerosis by Rubler et al. in 1972 [2]. Few clinical symptoms were observed in early DCM. However, with the further development of the disease, patients have become more susceptible to heart failure due to myocardial microvascular and metabolic disorders, which lead to changes in myocardial cell dysfunction and structure. Moreover, DCM is closely related to the high incidence of cardiovascular disease and high mortality in patients with diabetes. It was reported that the prevalence of DCM in patients with type 2 diabetes is approximately 12% [3].

At present, controlling blood sugar and improving heart failure are the main ways to overcome DCM. However, treatment with Western medicine remains unsatisfactory. Therefore, the diagnosis and therapy for DCM have presently become pressing problems. Traditional Chinese herbal formulation (TCMF) has been widely used in clinic due to its well-proven efficacy and few side effects. The Qing-Hua-Yu-Re-Formula (QHYRF) is a new herbal preparation for treating DCM, which was developed by Professor Wang Xu, according to the clinic experience of Chinese Medicine Master Professor Zhou Zhongying. The recipe of QHYRF comprises six herbal medicines: radix rehmanniae recen, *Salvia miltiorrhiza* Bge, cortex moutan, *Rhizoma coptidis*, radix paeoniae rubra, and *Euonymus alatus*. Emerging evidences have indicated that the six major active components (danshensu, paeoniflorin, acteoside, lithospermic acid, salvianolic acid B, and salvianolic acid C) in QHYRF protect cardiomyocytes by antioxidation, anti-inflammation, antiapoptosis, and decreasing calcium overload [4–7]. Moreover, clinical studies have shown that QHYRF could effectively improve symptoms in patients with DCM, including blood sugar, blood rheology, and left ventricular structure and function. According to the known effective components, we speculate that the effect of myocardial protection of QHYRF could be achieved through the following methods: (1) increase myocardial glucose transporter gene expression to improve glucose and lipid metabolism; (2) decrease the level of inflammatory cytokine hypersensitive C-reactive protein and tumor necrosis factor-alpha, and increase serum adiponectin levels to reduce inflammation; (3) reduce glucose toxicity; (4) increase insulin sensitivity and improve insulin resistance; (5) reduce the apoptosis of cardiac muscle cells and decrease the PAI-1 level of the fibrinolysis system; (6) improve blood stasis [8, 9].

It has been widely accepted that the chemical composition of traditional Chinese medicine (TCM) is complex, and its improper use may cause toxic effects [10]. Therefore, the quality control of TCM is extremely important. However, few researches have been carried on the chemical composition of QHYRF. Furthermore, the specific content of its main ingredients remains unknown. It should be noted that the constituents and contents of the main active components of QHYRF may be influenced by harvest time, plant origin, and manufacturing procedures [10, 11]. Thus, there is an urgent need to develop an effective method for QHYRF quality control, in order to guarantee its pharmacological efficacy.

In the present study, both qualitative and quantitative approaches were developed for the comprehensive quality control of QHYRF. Twenty-seven compounds were identified or tentatively characterized by ultrahigh-performance liquid chromatography coupled with quadrupole-time-of-flight mass spectrometry (UHPLC-Q-TOF/MS), including 12 phenolic acids, nine monoterpene glycosides, two flavonoids, three iridoids, and one unknown compound. Moreover, a simple, reliable, and sensitive analytical method, the high-performance liquid chromatography with ultraviolet detection (HPLC-UV) method, was used to determine the quantity of the six major active components (danshensu, paeoniflorin, acteoside, lithospermic acid, salvianolic acid B, and salvianolic acid C) of QHYRF. The potential application of the present study not only supports the quality control of QHYRF but also provides a theoretical research basis for the further research of QHYRF in clinic.

## 2. Experimental

### 2.1. Chemicals and Materials

Six crude herbs (cortex mouta, *Rhizoma coptidis*, *Euonymus alatus*, *Rehmannia glutinosa*, *Salvia miltiorrhiza,* and *Paeonia lactiflora*) were obtained from the pharmacy in Nanjing Hospital of Traditional Chinese Medicine, according to the Chinese Pharmacopoeia (2010 edition). The standard compounds (purity > 98%; danshensu, paeoniflorin, acteoside, lithospermic acid, salvianolic acid B, and salvianolic acid C) were purchased from Sichuan Victor Biotechnology Co., Ltd. HPLC-grade acetonitrile was obtained from Merck Co. All solutions and dilutions were prepared with ultrapure water obtained from a Milli-Q water purification system.

The extraction procedures for QHYRF total fraction were as follows: first, 15 g of radix rehmanniae recen, 15 g of *Salvia miltiorrhiza* Bge, 10 g of paeonol, 5 g of *Rhizoma coptidis*, 10 g of radix paeoniae rubra, and 10 g of *Euonymus alatus* were extracted twice with water (×8) to yield a crude extract. Second, the crude extract was concentrated and spray-dried to obtain the final product.

### 2.2. Sample Preparation

The samples were prepared as follows: 20 mg of QHYRF total fraction was dissolved in 1 mL of methanol and ultrasonicated for 15 minutes. Then, the sample solution was centrifuged at 12,000 rpm for 10 minutes, transferred to vials, and subjected to UHPLC-Q-TOF/MS and HPLC-UV analysis.

### 2.3. UHPLC-Q-TOF/MS Analysis

The analytical included a Shimadzu UHPLC system and a Q-TOF 5600-plus mass spectrometer equipped with Turbo V source and a TurboIonSpray interface (AB Sciex) and an Agilent 1100 LC-UV system with ChemStation (Agilent). The chromatographic conditions were as follows [12]: Kromasil C_18_ column (4.6 × 150 mm, 5 *μ*m) at 35°C, sample injection volume, 10 *μ*L; mobile phases, water containing 0.1% formic acid (solvent A) and acetonitrile (solvent B); gradient program was employed according to the following programmer: 0–5 minutes, 5–15% B; 5–10 minutes, linear increase to 20% B; 10–20 minutes, linear increase to 25% B; 20–30 minutes, linear increase to 100% B; 30–35 minutes, hold on 100% B.

The operating parameters for Q-TOF/MS were set as follows [12]: ion spray voltage, −4.5 kV; collision energy, −35 eV; nebulizer gas (gas 1), 55 psi; declustering potential, −60 V; heater gas (gas 2), 55 psi; turbo spray temperature, 550°C; curtain gas, 35 psi; resolution, 20,000. Full-scan data acquisition was performed from *m/z* 100 to 1500 in the negative mode.

### 2.4. HPLC-UV Analysis

The analysis was performed on a Shimadzu HPLC system. Chromatographic separation was achieved on an XBridge C_18_ column (4.6 × 150 mm, 5 *μ*m). The temperature of the column oven was set at 30°C, and the flow rate was 1.0 mL/min. The sample injection volume was 10 *μ*L. The mobile phases were a mixture of water with 0.1% formic acid (solvent A) and acetonitrile (solvent B). A gradient program was employed according to the following profile: 0–10 minutes, 10–15% B; 10–25 minutes, linear increase to 30% B; 25–60 minutes, linear increase to 100% B. The UV wavelength was set at 254 nm (paeoniflorin, lithospermic acid, and salvianolic acid B), 280 nm (danshensu and salvianolic acid C), and 320 nm (acteoside).

### 2.5. Validation of the Established HPLC-UV Approach

The stock solution that contained the six reference compounds (0.5 mg/mL danshensu, 25 mg/mL paeoniflorin, 1 mg/mL acteoside, 1 mg/mL lithospermic acid, 10 mg/mL salvianolic acid B, and 1 mg/mL salvianolic acid C) was prepared in methanol and stored at 4°C. In order to establish the calibration curves, the stock solution was diluted to appropriate concentrations. Solutions that contained different concentrations of the six standard compounds were injected in triplicate. The calibration curves were peak areas versus the concentration for each compound [12].

The limit of detection (LOD) was determined as a signal-to-noise ratio equal to 3, and the limit of quantification (LOQ) was based on 10 times of the signal-to-noise ratio value.

The precision was evaluated by intraday and interday variability. Intraday reproducibility was carried out by analyzing the individual sample solution six times within one day. For interday variability, six samples were determined six times in three consecutive days.

A stability study was performed with a sample solution checked at 0, 4, 8, 12, and 24 hours. Variations were expressed by relative standard deviations (RSDs).

Recovery studies were carried out by spiking known amounts of the reference compounds at low, medium, and high concentration in the samples. Then, the spiked samples were thoroughly mixed, extracted, and analyzed in accordance with the methods mentioned above. Recovery (%) = (amount found − original amount)/amount spiked × 100% [12].

### 2.6. Application to Different Batches of QHYRF

The established HPLC-UV method was subsequently employed for the simultaneous quantification of danshensu, paeoniflorin, acteoside, lithospermic acid, salvianolic acid B, and salvianolic acid C in QHYRF from 10 different batches.

## 3. Results and Discussion

### 3.1. UHPLC-Q-TOF-MS Analysis

**Figure 1:**
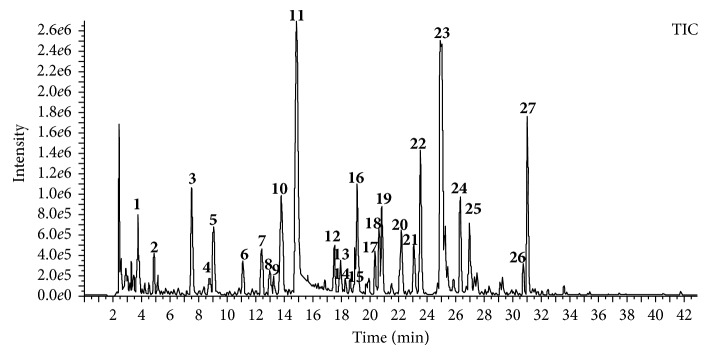
TIC chromatogram of QHYRF using UHPLC-Q-TOF/MS.

The UHPLC-Q-TOF/MS approach was employed for the separation and identification of compounds in QHYRF. The total ion current chromatogram is shown in Figure 1. A total of 27 compounds were identified, including 12 phenolic acids, nine monoterpene glycosides, two flavonoids, three iridoids, and one unknown compound. The detailed information is displayed in Table 1. The retention time and fragmentation information of compounds **2**, **11**, **16**, **22**, **23**, and **26** were compared with that of the standard compounds.

**Figure 2:**
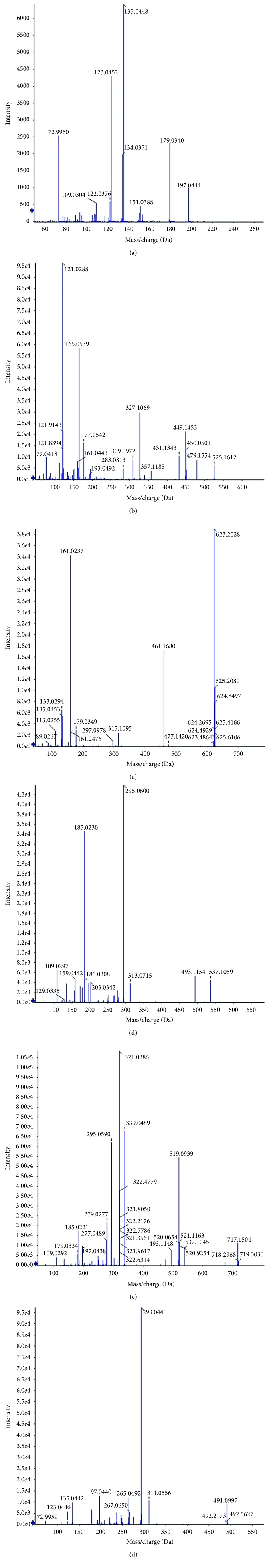
MS^2^ spectra of peak 2 (a), peak 11 (b), peak 16 (c), peak 22 (d), peak 23 (e), and peak 26 (f) in the QHYRF.

Phenolic acids were the major components of QHYRF. A total of 12 phenolic acids were identified and tentatively characterized based on their mass data and reports in literature. These always exhibited a unique fragmentation behavior. The characteristic mass behaviors of these phenolic compounds were the losses of danshensu (198 Da) and caffeic acid (180 Da) molecules. Peak **2** revealed the [M-H]^−^ ion at *m/z* 197.0467 and gave the element composition of C_9_H_10_O_5_. In the MS^2^ spectra, the ions at *m/z* 135 and 179 were observed, which was consistent with that of danshensu. In comparison with the reference, compound **2** was unambiguously identified as danshensu (Figure 2(a)). Peaks **20**, **23**, and **24** displayed the same [M-H]^−^ ion at *m/z* 717.1472 (C_36_H_30_O_16_). In comparison with standard compound, compound **23** was unambiguously assigned as salvianolic acid B (Figure 2(e)). The product ion at *m/z* 339 was formed due to the disconnection of another caffeic acid (180 Da) molecule from the deprotonated daughter ion [M-H-198]^−^ at *m/z* 519. Further loss of H_2_O led to the yield of product ion at *m/z* 321. Peaks **20** and **24** shared the same MS^2^ ions with that of compound **23**. Compared with literatures [13, 14], compounds **20** and **24** was plausibly deduced as salvianolic acid E and isosalvianolic acid B. Peaks **14**, **18**, and **22** were a group of isomeric compounds, and these displayed [M-H]^−^ ions at *m/z* 537.1041 (C_27_H_22_O_12_). These had similar fragmentation pathways to those of salvianolic acid A and generated similar fragment ions at *m/z* 313, 295, and 185. Peak **22** was unequivocally identified as lithospermic acid by comparison with the standard (Figure 2(d)). According to the literature [14], peaks **18** and **14** were tentatively identified as salvianolic acid H or salvianolic acid A. Peak **19** revealed the [M-H]^−^ ion at *m/z* 417.0826 and gave the element composition of C_20_H_18_O_10_. This generated a series of fragment ions at *m/z* 373, 197, 179, and 175. Compared with the literature [15], compound **19** was tentatively deduced as salvianolic acid D. Peak **21** displayed the [M-H]^−^ ion at *m/z* 359.0775 and produced fragment ions at *m/z* 197, 179, and 161. In comparison with the reference [15], compound **21** was plausibly assigned as rosmarinic acid. Peak **26** gave an [M-H]^−^ ion at *m/z* 491.0983 with the molecular composition C_26_H_20_O_10_. Compared with the standard, peak **26** was unequivocally identified as salvianolic acid C (Figure 2(f)). Peaks **5** and **10** displayed the same [M-H]^−^ ion at *m/z* 367.1033 with the molecular composition C_17_H_20_O_9_. These two compounds yielded similar product ions at *m/z* 193 and 134. Compared with literatures [16, 17], compounds **5** and **10** were tentatively assigned as 5-O-feruloylquinic acid and 3-O-feruloylquinic acid, respectively.

Monoterpene glycosides exhibited quasi-molecular ions [M-H]^−^ and [M-H + HCOO]^−^ in the negative ion mode. The chemical structure of aglycones is a “cage-like” pinane skeleton, which is very unusual among natural products. The monoterpene glycosides in QHYRF were usually esterified with an aromatic acid such as benzoic acid, vanillic acid, methoxybenzoic acid, and gallic acid. A total of nine monoterpene glycosides were identified. Peaks **4**, **11**, and **15** displayed the same [M-H]^−^ ion at *m/z* 525 with the molecular composition C_24_H_30_O_13_. Peaks **11** and **15** produced the same product ions at *m/z* 479, 449, and 121. In comparison with the standard, peak **11** was unambiguously identified as paeoniflorin (Figure 2(b)). Peak **15** was plausibly assigned as the paeoniflorin isomer. Peak **4** yielded the product ions at *m/z* 495 and 167. In comparison with the literature [18], peak **4** was deduced as mudanpioside E. Peak **3** gave the [M-H]^−^ ions at *m/z* 495.1498 (C_23_H_28_O_12_) and was 16 Da (O) more than that of paeoniflorin. It produced fragment ions at *m/z* 465.1437 ([M-H-HCOH]^−^), indicating that peak **3** may be oxypaeoniflorin based on a previously reported literature data [13]. Peaks **6** and **7** displayed the same [M-H]^−^ ions at *m/z* 505.1562 (C_20_H_28_O_12_) and generated similar product ions at *m/z* 459, 293, and 150. Compared with literatures [19], peaks **6** and **7** were tentatively deduced as paeonolide and apiopaeonoside, respectively. Peak **8** revealed the [M-H]^−^ ion at *m/z* 647.1615, which was 152 Da (galloyl group, C_7_H_4_O_4_) more than that of peak **3**. This produced fragment ions at *m/z* 629, 509, and 491 by a series of losses of one H_2_O molecule, one benzoic acid molecule, and one H_2_O molecule, respectively. Compared with the literature [20], peak **8** was tentatively characterized as 6-O-galloyloxypaeoniflorin. Peak **25** gave the [M-H]^−^ ions at *m/z* 599.1769 (C_30_H_32_O_13_) and was 104 Da more than that of peak **3**. This produced fragment ions at *m/z* 569 and 477, indicating that peak **25** may be benzoyloxypaeoniflorin, based on a previously reported literature data [19]. Peak **27** revealed the [M-H]^−^ ions at *m/z* 629.1872 and was 104 Da more than that of paeoniflorin. Compared with the literature [16], peak **27** was tentatively characterized as benzoylpaeoniflorin.

Iridoids were also observed in the formula. Peaks **16** and **17** revealed the same [M-H]^−^ at *m/z* 623 and produced the same fragments at *m/z* 461 and 161. Compared with the standard, compound **16** was unambiguously identified as acteoside (Figure 2(c)). The retention time of peak **17** was a little later than that of peak **16**. Compared with the literature, compound **17** was tentatively deduced as forsythoside A. Peak **9** displayed the [M-H]^−^ ion at *m/z* 785.2520 with the molecular composition C_35_H_46_O_20_. This produced product ions at *m/z* 623 [M-Glc]^−^, 477 [M-Glc-Ara]^−^, and 179. In comparison with the literature, compound **9** was tentatively deduced as echinacoside. Peaks **12** and **13** revealed the same [M-H]^−^ ions at *m/z* 611.1621 and yielded product ions at *m/z* 445 and 169. Compared with the reference [21], compounds **12** and **13** were tentatively characterized as hydroxysafflor yellow A and its isomer.

### 3.2. Development and Validation of the HPLC-UV Approach

**Figure 3:**
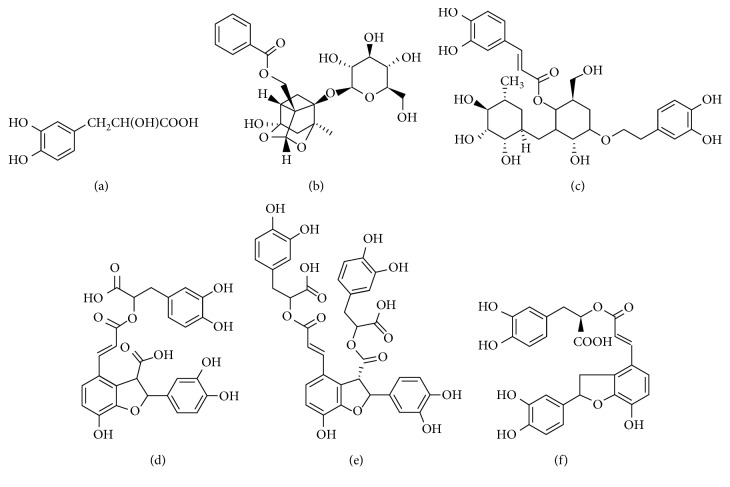
Chemical structure of danshensu (a), paeoniflorin (b), acteoside (c), lithospermic acid (d), salvianolic acid B (e), and salvianolic acid C (f).

The HPLC separation conditions were optimized including the mobile phase system, column temperature, and UV detection wavelength. In order to obtain chromatograms with better resolution of the adjacent peaks and shorter time, methanol and acetonitrile were compared in the experiment. The result indicated that acetonitrile was better than methanol due to shorter analysis time and better peak shape. In addition, the different column temperatures were also optimized. Finally, 30°C was chosen as the best column temperature. Under present HPLC conditions, the samples of QHYRF and standard solutions were analyzed. The UV absorption and detection wavelength of each compound were confirmed as follows: paeoniflorin, lithospermic acid and salvianolic acid B (254 nm), danshensu and salvianolic acid C (280 nm), and acteoside (320 nm) (chemical structure is shown in Figure 3).

**Figure 4:**
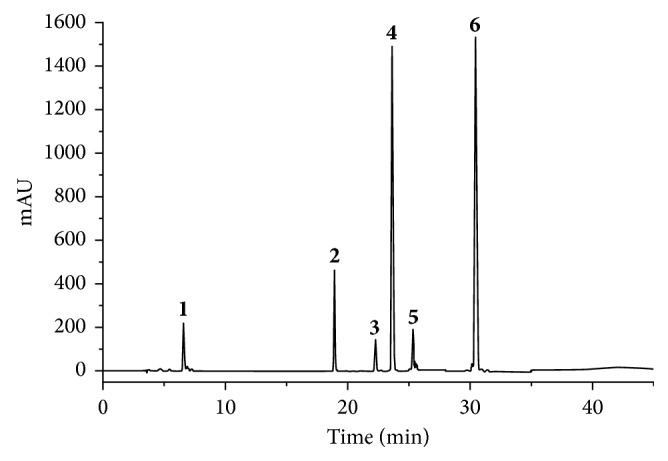
HPLC chromatogram of QHYRF using UV detector. Danshensu **(1)**, paeoniflorin **(2)**, acteoside **(3)**, lithospermic acid **(4)**, salvianolic acid B **(5)**, and salvianolic acid C **(6)**.

According to the representative chromatograms of the standard solution, sample solution, and negative control samples solution, it could be found that the determination of these six compounds does not interfere with each other (Figure 4).

The calibration curves of danshensu, paeoniflorin, acteoside, lithospermic acid, salvianolic acid B, and salvianolic acid C were linear within the range of 2.6–500, 20.8–25,000, 5.2–1000, 10.4–1000, 5.2–10,000, and 10.4–1000 *μ*g/mL, respectively. The correlation coefficient (*R*^2^) of all six standards revealed a good linearity of >0.991 in the aforementioned ranges. The LODs and LOQs of these six compounds were 0.6–5.0 and 2.6–20.8 *μ*g/mL, respectively (Table 2).

Intraday and interday variations were chosen to evaluate the precision of the method. These results are shown in Table 2. It was revealed that the intraday and interday RSDs were within the range of 1.6%–4.8% and 1.2%–2.2% (*n*=6). The stability of six major compounds ranged from 2.8% to 4.7%, which met the requirements of the analytical approach (Table 3).

The recovery data are shown in Table 4, which represents the accuracy of the method and was sufficient for the analysis. The measured data indicated that the recovery of these six components ranged from 99.0% to 104.7%.

### 3.3. Application

The established HPLC-UV approach was employed for simultaneously quantifying the six active components in 10 batches of QHYRF. These results are presented in Table 5. Furthermore, these results revealed that there were small differences among the contents of the six major constituents in different batches of QHYRF.

Optimized chromatographic conditions for the good resolution of adjacent peaks were achieved after several trials with elution systems of acetonitrile-water, methanol-water, acetonitrile-acid, and methanol-acid in different proportions. It was found that the presence of 0.1% fomic acid resulted in the significant improvement on the retention behavior of the different components. The optimal mobile phase, which consists of acetonitrile-0.1% fomic acid, was finally employed. This led to its high resolution and symmetrical peak shape.

According to the theory of TCM, QHYRF was prepared by six herbal medicines to cure the disease of diabetic cardiomyopathy. The presence of the researched active compounds in QHYRF was proved, making it possible to suggest a therapeutic effect in clinical applications. However, it enhances the complexity of the constituents and preparation procedures, which make it difficult to ensure the batch-to-batch uniformity of QHYRF. Thus, the quantitative measurement of its bioactive components is extremely necessary during the preparation and application of this prescription.

In recent years, methods for multicomponent analysis have become a credible solution for the analysis of a complex system in TCM. At present, we developed a sensitive HPLC-UV method to simultaneously quantify six active components in 10 different batches of QHYRF. A slight variation was observed in the different batches of QHYRF during the determination of the six major constituents, which reveal that the present preparation of QHYRF has good reproducibility. Furthermore, good reproducibility ensures the safety and effects of QHYRF in clinical application.

## 4. Conclusion

In summary, a simple and sensitive UHPLC-Q-TOF/MS method was established to qualitatively analyze the chemical components of QHYRF. Twenty-seven compounds were identified or tentatively characterized based on retention time, and MS^1^ and MS^2^ data, or by comparing with standards and literatures. These compounds include 12 phenolic acids, nine monoterpene glycosides, two flavonoids, three iridoids, and one unknown compound. A HPLC-UV method for the simultaneous determination of six active components from QHYRF was developed and well validated, showing its excellent precision, stability, and recovery. In addition, these results indicate a slight variation in the contents of the six major constituents among the different batches of QHYRF. This is the first comprehensive study that qualitatively and quantitatively determined the chemical constituents of QHYRF using UHPLC-Q-TOF/MS and HPLC-UV. Thus, these findings could serve as a foundation for the quality control or further research of QHYRF.

## Figures and Tables

**Table 1:** MS^1^ and MS^2^ information of the QHYRF.

Number	*t*_*R*_ (min)	MS^1^	MS^2^	Formula	Error	Identification	Source
1	3.7	719.2063	**359.0976**, 197.0432, 179.0326	C_48_H_32_O_7_	−1.7	—	—
2^∗^	4.9	197.0467	179.0340, **135.0448**, 123.0452, 72.9960	C_9_H_10_O_5_	5.9	Danshensu	*Salvia miltiorrhiza*
3	7.5	495.1498	465.1405, 345.1118, 137.0228	C_23_H_28_O_12_	−2.0	Oxypaeoniflorin	*Paeonia lactiflora*
4	8.7	525.1612	495.1546, **167.0338**	C_24_H_30_O_13_	−0.3	Mudanpioside E	*Salvia miltiorrhiza*
5	9.0	367.1033	193.0490, 134.0362	C_17_H_20_O_9_	−0.4	5-O-Feruloylquinic acid	*Salvia miltiorrhiza*
6	11.1	505.1562	459.1522, 293.0870, **165.0543**, 150.0308	C_20_H_28_O_12_	3.0	Paeonolide	*Paeonia lactiflora*
7	12.4	505.1563	459.1526, 293.0873, 233.0656, **165.0547**, 150.0310	C_20_H_28_O_12_	0.0	Apiopaeonoside	*Salvia miltiorrhiza*
8	13.0	647.1615	629.1577, 509.1336, 491.1226,399.0946, 313.0565, **271.0453**	C_30_H_32_O_16_	−0.4	6-*O*-Galloyloxypaeoni- florin	*Paeonia lactiflora*
9	13.3	785.252	**623.2223**, 477.1619, 179.0345, 161.0227	C_35_H_46_O_20_	1.3	Echinacoside	*Rehmannia glutinosa*
10	13.8	367.1036	193.0487, 191.0543, **173.0439**, 134.0362	C_17_H_20_O_9_	0.4	3-O-Feruloylquinic acid	*Salvia miltiorrhiza*
11^∗^	14.8	525.1608	479.1554, 449.1453, 327.1069, 165.0539, **121.0288**	C_24_H_30_O_13_	−1.1	Paeoniflorin	*Paeonia lactiflora*
12	17.5	611.1621	**445.0995**, 343.0670, 169.0124	C_27_H_32_O_16_	0.6	Hydroxysafflor yellow A	*—*
13	17.9	611.1621	**445.0998**, 169.0131	C_27_H_32_O_16_	0.6	Hydroxysafflor yellow A isomer	*—*
14	18.3	537.1041	493.1154, 313.0515, **295.0600**, 185.0230	C_27_H_22_O_12_	0.5	Salvianolic acid A	*Salvia miltiorrhiza*
15	18.6	525.16	479.1589, 449.1472, 317.1086, 283.0818, **121.0293**	C_24_H_30_O_13_	−0.1	Paeoniflorin isomer	*Paeonia lactiflora*
16^∗^	18.9	623.1979	461.1680, **161.0237**	C_29_H_36_O_15_	−0.4	Acteoside	*Rehmannia glutinosa*
17	20.3	623.1983	461.1683, **161.0234**	C_29_H_36_O_15_	0.2	Forsythoside A	*Rehmannia glutinosa*
18	20.6	537.1034	493.1155, 313.0502, **295.0594**, 185.0226	C_27_H_22_O_12_	−0.8	Salvianolic acid H	*Salvia miltiorrhiza*
19	20.8	417.0826	373.0918, 197.0432, 179.0328, **175.0379**, 152.0279	C_20_H_18_O_10_	−0.3	Salvianolic acid D	*Salvia miltiorrhiza*
20	22.2	717.1472	519.0950, 339.0500, **321.0395**, 295.0597	C_36_H_30_O_16_	1.5	Salvianolic acid E	*Salvia miltiorrhiza*
21	23.1	359.0775	197.0442, 179.0334, **161.0233**, 133.0287	C_18_H_16_O_8_	0.7	Rosmarinic acid	*Salvia miltiorrhiza*
22^∗^	23.5	537.1037	493.1172, 313.0509, **295.0608**, 185.0236	C_27_H_22_O_12_	−0.3	Lithospermic acid	*Salvia miltiorrhiza*
23^∗^	24.9	717.1456	519.0939, 339.0489, **321.0386**, 295.0590, 279.0277	C_36_H_30_O_16_	−0.7	Salvianolic acid B	*Salvia miltiorrhiza*
24	26.3	717.1468	519.0936, 339.0487, **321.0385**, 295.0588, 279.0275	C_36_H_30_O_16_	1.0	Isosalvianolic acid B	*Salvia miltiorrhiza*
25	27.0	599.1769	569.1709, 477.1430, **137.0234**	C_30_H_32_O_13_	−0.2	Benzoyloxypaeoniflorin	*Paeonia lactiflora*
26^∗^	30.7	491.0983	311.0556, **293.0440**, 265.0492, 197.0440, 135.0442	C_26_H_20_O_10_	−0.1	Salvianolic acid C	*Salvia miltiorrhiza*
27	31.0	629.1872	583.1838, 553.1730, 431.1342, 165.0541, **121.0287**	C_30_H_32_O_12_	−0.6	Benzoylpaeoniflorin	*Paeonia lactiflora*

^∗^Indicated compared with standards. The bold numbers represent the most abundant ions.

**Table 2:** Linear regression data, LODs, and LOQs of six compounds.

Components	Regression equations	*R* ^2^	Linear range (*μ*g/mL)	LODs (*μ*g/mL)	LOQs (*μ*g/mL)
Danshensu	*y* = 5508.1*x* + 5.404	0.9999	2.6–500	0.7	2.6
Paeoniflorin	*y* = 419.45*x* − 6.7219	0.9994	20.8–25,000	5.0	20.8
Acteoside	*y* = 3283.3*x* − 11.072	0.9997	5.2–1000	1.0	5.2
Lithospermic acid	*y* = 12900*x* − 123.54	0.9991	10.4–1000	0.6	10.4
Salvianolic acid B	*y* = 1616.6*x* − 9.4854	0.9993	5.2–10,000	2.0	5.2
Salvianolic acid C	*y* = 19071*x* + 88.12	1.0000	10.4–1000	0.8	10.4

*y* = *Ax* + *B*; *y* is the peak area; *x* is the concentration of the analytes; *R*^2^ is the correlation coefficient of the equation.

**Table 3:** Precision and stability of six compounds in QHYRF (*n*=6).

Components	Precision		Stability RSD (%)
	Intraday RSD (%)	Interday RSD (%)	
Danshensu	3.8	2.2	4.7
Paeoniflorin	1.6	1.7	4.2
Acteoside	4.3	1.2	2.8
Lithospermic acid	4.1	1.6	2.8
Salvianolic acid B	4.8	2.1	2.4
Salvianolic acid C	4.7	1.2	3.9

**Table 4:** Recovery of six compounds in QHYRF (*n*=3).

Components	Contents	Quantity added	Theoretical amount	Recorded amount	Recovery	RSD
	(mg/mL)	(mg/mL)	(mg/mL)	(mg/mL)	(%)	(%)
Danshensu	0.22	0.05	0.27	0.27	100.6	1.7
		0.11	0.33	0.33	100.0	3.3
		0.2	0.42	0.42	100.2	1.8
Paeoniflorin	12.32	2.45	14.77	14.78	100.4	0.4
		4.95	17.27	17.27	100.0	2
		7.85	20.17	20.17	100.0	5.4
Acteoside	0.07	0.15	0.22	0.22	103.1	3.7
		0.22	0.29	0.29	99.0	5.5
		0.42	0.49	0.49	100.6	5.3
Lithospermic acid	0.1	0.08	0.18	0.18	102.6	4.3
		0.15	0.25	0.25	103.3	5.4
		0.27	0.37	0.38	102.2	1.7
Salvianolic acid B	4.96	1	5.96	5.96	99.9	3.7
		2.23	7.19	7.19	100.0	3.5
		3.41	8.37	8.37	100.1	2.2
Salvianolic acid C	0.01	0.09	0.1	0.10	103.6	3
		0.21	0.22	0.22	99.0	2.3
		0.35	0.36	0.38	104.7	4.8

**Table 5:** Content of six components in 10 batches of the QHYRF.

Components	Content (mg/g)									
	1	2	3	4	5	6	7	8	9	10
Danshensu	9.8	10.0	9.8	10.0	10.0	9.8	10.1	10.2	10.0	9.7
Paeoniflorin	582.6	591.2	612.4	579.8	617.3	591.4	609.3	582.4	582.0	601.4
Acteoside	3.4	3.4	3.3	3.4	3.3	3.3	3.5	3.2	3.4	3.4
Lithospermic acid	4.5	4.7	4.6	4.4	4.5	4.6	4.6	4.5	4.6	4.6
Salvianolic acid B	265.9	266.4	260.5	260.7	263.4	262.6	272.8	263.5	264.8	270.7
Salvianolic acid C	0.5	0.5	0.5	0.5	0.5	0.5	0.5	0.5	0.5	0.5
